# The associations between ATN biomarkers and episodic memory diminish as age increases

**DOI:** 10.1007/s41999-026-01606-8

**Published:** 2026-09-08

**Authors:** Urban Ekman, Måns Gyllenhammar, Ove Almkvist, Anton Ocklind, Leo Hallerstam, Daniel Ferreira, Maria Eriksdotter, Lars-Olof Wahlund, Anna Rennie, Eric Westman

**Affiliations:** 1 Division of Clinical Geriatrics, Department of Neurobiology, Care Sciences and Society, Center for Alzheimer Research, Karolinska Institutet, Stockholm, Sweden; 2 Medical Unit Allied Health Professionals, Women’s Health and Allied Health Professionals Theme, Karolinska University Hospital, Stockholm, Sweden; 3 Facultad de Ciencias de la Salud, Universidad Fernando Pessoa Canarias, Las Palmas, Spain; 4 Theme Aging, Karolinska University Hospital, Stockholm, Sweden; 5 The Ageing Epidemiology Research Unit, School of Public Health, Imperial College London, London, UK; 6 Stockholm, Sweden

**Keywords:** Episodic memory, Cognitive disorders/dementia, Cognitive aging, CSF, Structural brain imaging, Cognitive diagnostics

## Abstract

**Purpose:**

The relationship between age, biomarkers, and cognitive function at advanced ages remains underexplored. By utilizing a naturalistic memory clinic cohort, we aimed to clarify how the A/T/N biomarkers are associated with verbal episodic memory performance and the moderating effect of age.

**Methods:**

This cross-sectional study included 676 patients from memory clinics in the Stockholm metropolitan region, comprised of 141 with Alzheimer’s disease dementia, 403 with mild cognitive impairment, and 132 with subjective cognitive impairment. We used weighted least-squares regressions to evaluate the association between episodic memory and the biomarkers and the moderating effect of age.

**Results:**

There was a significant interaction between age and A (Aβ42), where the negative impact of Aβ42 abnormality on Rey Auditory Verbal Learning (RAVLT) delayed recall was reduced with higher age (*β* = 0.22, *p* < 0.001). In addition, a corresponding interaction effect was observed between age and N (MTA) on RAVLT delayed recall (*β* = 0.13, *p* < 0.01). Additional exploration analyses showed that at age 80 or older, the ATN biomarkers showed weaker associations with episodic memory performance.

**Conclusions:**

Although interpretations should be conducted with caution, these findings raise questions about the justification for assessing CSF in patients older than 80 years, given its weak associations with core clinical measures, such as learning and free recall of episodic memory. Further clinical studies are needed to validate these results and also evaluate other core cognitive measures. The naturalistic nature of the memory clinic population, with its clinically driven diagnoses, may help clarify these findings.

**Supplementary Information:**

The online version contains supplementary material available at https://doi.org/10.1007/s41999-026-01606-8.

## Key summary points

**Aim:**

This study aimed to clarify the links between amyloid-β42 (A), phosphorylated tau (T), and medial temporal atrophy (N) with verbal episodic memory performance, as well as to explore the moderating effect of age on these relationships.

**Findings:**

Our findings revealed significant interaction effects of age between amyloid-β42 (A) and medial temporal atrophy (N) in relation to verbal episodic memory recall. Specifically, the detrimental influence of Aβ42 and MTA abnormalities on episodic memory performance was attenuated with advancing age.

**Message:**

The primary findings suggest that the association between ATN biomarkers and episodic memory is attenuated as age increases, which may have clinical implications.

**Supplementary Information:**

The online version contains supplementary material available at https://doi.org/10.1007/s41999-026-01606-8.

## Background

Humans undergo various changes as they age, leading to increased challenges for the cognitive diagnostic procedure. These changes encompass the cognitive aging process [1], and reflect the heterogeneous nature of aging [2]. To manage this heterogeneity, biomarkers have become increasingly central [3]. The National Institute on Aging and Alzheimer’s Association (NIA-βAA) [4] and the International Working Group (IWG) [5] agreed upon the critical role of biomarkers in supporting the diagnosis of Alzheimer’s disease (AD). Increased focus on biomarkers in diagnostic procedures is reflected in the A/T/N model [6]. The letter ‘A’ stands for Amyloid Beta values, ‘T’ for phosphorylated tau, and ‘N’ for neurodegeneration. The brain neuropathological background for this model is the presence of extracellular plaques primarily composed of beta-amyloid (Aβ) and intracellular neurofibrillary tangles (NFTs) containing tau, the two hallmarks of AD [7]. However, although biomarkers have become increasingly important in both research and clinical care, their use and interpretation are not without controversy due to a lack of standardization and the risk of overdiagnosis [8], and the traditional clinical symptom-based approach remains widely used. In clinical care, a combination of biomarkers assessed through neuroimaging and cerebrospinal fluid (CSF) samples is commonly used, but clinical symptoms remain essential for accurate diagnosis [3]. Moreover, the relationship between biomarkers, cognitive function, and aging is not fully understood, particularly at older ages [9]. Regarding the relationship between biomarkers and cognition in older age groups, a review of the sensitivity of biomarkers in AD diagnostics showed weak evidence for the usefulness of these biomarkers in the oldest old (≥ 85 years) [9]. Thus, some studies indicate that the associations between biomarkers and cognition may decrease with advancing age. Additionally, an autopsy study found a strong association between AD-pathological changes and dementia at 75 years of age, but a substantially lower association at 95 years of age [10]. However, whether this trend is observed in all populations and contexts, as well as across all cognitive domains, remains unclear. Lower biomarker accuracy is expected in the older population due to multiple pathologies, due to multimorbidity, and conditions that mimic the clinical characteristics of AD [9], such as impairments in learning ability and episodic memory recall. Additionally, AD pathology may have no clinical implications because it remains in a preclinical phase.

Episodic memory impairment is a central hallmark of typical AD and one of the cognitive functions most severely affected in early clinical stages. Although individual differences exist in the encoding and retrieval of episodic memory with advancing age, an age-related decline in function can generally be observed from age 60, with older adults typically showing a decrease in episodic memory function [11]. Episodic memory learning (encoding) and episodic memory free recall are critical in relation to clinical biomarker changes, especially when evaluating the clinical application of these biomarkers as individuals age. Aging and co-occurring pathologies may modify the clinical expression of AD biomarkers, leading to weaker biomarker-cognition associations in later life, and the negative association between ATN abnormalities and episodic memory performance could be attenuated with increasing age [12]. As a clinical measure of episodic memory, this study will utilize the Rey Auditory Verbal Learning Test (RAVLT), as RAVLT was the most frequently used episodic memory measure across the participating memory clinics, making it the most appropriate choice of outcome measure for the present study. As a measure of episodic memory, RAVLT is a test with strong clinical utility and widespread use in cognitive diagnostics [13]. For example, individuals diagnosed with MCI and characterized by A+/T+/N+ biomarkers have demonstrated that RAVLT performance is associated with alterations in brain network activity and structure [14], and amyloid positivity on the AD continuum has been associated with impaired performance in RAVLT [15]. We have also shown that RAVLT is associated with medial temporal atrophy [16], and is valid in differentiating levels of cognitive impairments [17, 18]. One general limitation of studies concerning biomarkers and cognition in the field of cognitive impairment is that they rely on selected research populations [19]. To enhance ecological validity, we analyzed data from the naturalistic MemClin cohort [20] of individuals assessed due to cognitive complaints. The moderating effect of age on the association between ATN biomarkers and cognitive performance remains insufficiently explored. We propose that the naturalistic character combined with the ATN classification framework will contribute valuable insights into age-related moderation effects, with potential implications for clinical practice.

This study aimed to clarify the associations of A (Aβ42), T (p-tau), and N (medial temporal atrophy, MTA) with verbal episodic memory measures and the moderating effect of age. When measuring episodic memory learning (encoding) and episodic memory delayed free recall, we expected to find interaction effects between the biomarker values and age. Specifically, we anticipated that the negative impact of biomarker abnormalities on these episodic memory functions would decrease with increasing age.

## Methods

This cross-sectional study recruited patients from the MemClin project, covering 9 out of 10 memory clinics in the Stockholm metropolitan region [20]. From 2416 patients, we excluded those with conditions other than SCI, MCI, AD, or Mixed Dementia (i.e., AD plus Vascular Dementia), leaving 2227 patients. We refer to the dementia group in the current study as AD. Of those 2227, 1486 had complete RAVLT data. We also excluded participants with incomplete information on age, sex, or years of education. Of those remaining, 676 patients had complete CSF data (Aβ42, p-tau). Consequently, the final sample consisted of 676 patients (AD = 141, MCI = 403, and SCI = 132). We included patients with complete data except for MTA, for which 587 patients had available data. To clarify the reasons for the missing data, we would like to point out that each participating memory clinic, physicians and psychologists independently determine which assessments they consider clinically relevant to administer. Consequently, not all participants underwent CSF biomarker assessment or completed the RAVLT. In addition, some data were unavailable due to administrative challenges.

### Diagnostic procedures

Details on the clinical examination have been described previously [20]. In brief, they followed the Swedish Board of Health and Welfare (2010; revised in 2017) [21]. All patients referred for a neuropsychological examination were asked to participate, giving the study a naturalistic character. Patients with subjective cognitive impairment (SCI) showed no objective cognitive impairment, in line with recent research and World Health Organization guidelines [22]. Mild cognitive impairment (MCI) was diagnosed using international consensus criteria [23], including subjective symptoms and objective decline without meeting dementia criteria. Objective findings were confirmed through neuropsychological assessments, adjusted for premorbid ability. Dementia diagnoses followed the International Classification of Diseases (ICD)− 10 and 11 criteria [22, 24]. All diagnoses were determined through multidisciplinary consensus meetings, based on comprehensive clinical examinations. The diagnosis was primarily driven by the clinical picture, with biomarkers providing supportive evidence. Thus, as the diagnoses are clinical, biomarkers are supportive in the clinical context rather than a prerequisite.

### Cognitive assessments

Episodic memory learning (the initial encoding of information) and episodic memory delayed recall (the ability to access learned information) were assessed using the RAVLT 15-item word list, using the standard procedure [25, 26]. Learning was scored as the total of all five trials, ranging from 0 to 75, and delayed recall was scored as the number of words recalled after a 30-min delay, with scores ranging from 0 to 15.

### Assessment of the A/T/N classification system

The A/T/N is a three-tailed binary +/− categorization system. We categorized the “A” biomarker with CSF Amyloid Beta 42 peptide (Aβ42), the “T” biomarker with CSF phosphorylated tau (p-tau), and the “N” biomarker with MTA derived from either CT or MRI. Each included individual was rated as positive (+ ; i.e., abnormal) or negative (− ; i.e., normal) on each biomarker as explained in the next section.

### Classification of CSF markers

The clinics transfer their CSF samples to two different laboratories for analysis. During the data collection period, the laboratories transitioned from the earlier Enzyme-Linked Immunosorbent Assay (ELISA) method to Electrochemiluminescent Immuno Assay (ECLIA) methods. ELISA and ECLIA have different cut-off values to differentiate normal from abnormal, and, therefore, prevented us from using CSF as a continuous variable. In the current sample, the cut-off values used were the same as those used at the clinics. Measurements taken with the ELISA method were considered abnormal if < 550 ng/l for Aβ42 and > 80 ng/l for p-tau. ECLIA's corresponding cut-off values were < 599 ng/l for Aβ42 and > 56.5 ng/l for p-tau [27].

### Classification of medial temporal lobe atrophy

Brain imaging was performed at various centers in Stockholm, using either a high-resolution CT scan or an MRI examination. In total, 60% of the patients received a CT scan, and 40% an MRI examination. Since visual ratings on CT and MRI are comparable, we combined them for analysis [28]. Radiologist rated medial temporal atrophy (MTA; scored from zero to four) using the Scheltens scale [29]. Cutoffs followed Ferreira et al. [30], and an MTA score ≥1.5 was considered abnormal in the age group 65–74, ≥ 2 for the age group 75–84, and ≥ 2.5 for the age group 85–94.

### Statistical analysis

SPSS version 29.0 was used for statistical analyses. The descriptive analyses were performed with one-way ANOVA and chi-squared analysis.

The models were evaluated to verify compliance with key statistical assumptions, including linearity, normality, homoscedasticity, and independence of residuals, to ensure the robustness and validity of the model estimates. Visual inspection revealed unequal variance on the dependent episodic memory variables (i.e., several scored zero on delayed recall). Scoring zero on this test condition is expected, given that the samples consist of memory clinic patients, many of whom are diagnosed with either MCI or dementia, and it is likely to be a somewhat reliable estimation of their episodic memory function. Therefore, the disproportionate number of zeroes is because there is a greater number of “true zeroes”, and not because the variable is composed of count data where non-zero values are rare, and zeroes might be inflated. Therefore, we used weighted least squares (WLS) regression to evaluate if the relationship between episodic memory and biomarkers is moderated by age. The absolute residuals were regressed on the predicted values (standard deviation function) to estimate the weights before we performed the WLS. The episodic memory measures (RAVLT total learning and delayed free recall) were used as dependent variables in six multiple linear regression models. Because the Aß_42_, p-tau, and MTA values were assessed with different methods and cut-offs (see above), those variables are included as binary, i.e., 0 or 1, reflecting normal and abnormal patterns. Each regression model was hierarchically built: In the first step (1), cognitive measure = B0 + B1*Gender + B2*Education + B3*Biomarker + B4*Age. In the second step (2), an interaction factor (Age*Biomarker) was added to the model, cognitive measure = B0 + B1*Gender + B2*Education + B3*Biomarker + B4*Age + B5*(Age*Biomarker). To more precisely examine and quantify the age at which abnormal Aβ42 stops having a significant effect on episodic memory performance, the relationship between abnormal Aβ42 status and episodic memory performance was regressed multiple times, stratified by age. Given sample sizes, age was stratified by two-year spans to balance statistical power with the precision of potential age cut-offs. The models were controlled for gender and years of education.

Given that six regression models were tested, we used a Bonferroni correction to adjust for the number of comparisons (calculated as 0.05 divided by 6, resulting in p < 0.008).

## Results

Table 1 describes the participants' demographics, cognitive performances, and biomarker abnormalities. The information is presented for the total sample and is separated by diagnosis (SCI, MCI, and AD dementia). Although 41.84% of the AD patients had normal Aβ42 markers and 45.39% had normal p-tau markers according to the clinical cut-offs, several patients had values close to the cut-offs (see Supplementary Fig. 1 for an illustration of Aβ42 and p-tau levels). The ages ranged from 35.92 to 94.29 years.

**Table 1:** Demographics and clinical characteristics

	Total (*n* = 676)	SCI (*n* = 132)	MCI (*n* = 403)	Dementia (*n* = 141)	*p*-value and effect size
Years of education	13.1 (13.14)	14.13 (3.71)	13.07 (3.56)	12.40 (3.90)	*P* < 0.001 *n*^2^ = 0.02
Age	73.26 (8.49)	66.76 (9.80)	74.89 (7.12)	74.72 (7.88)	*P* < 0.001 *n*^2^ = 0.14
Gender number of females, males	362, 314	88, 48	205, 203	76, 68	*P* = 0.48, Cohen’s W = 0.01
*Cognitive performance*					
MMSE	26.90 (2.61)	28.31 (2.36)	27.25 (2.10)	24.65 (2.87)	*P* < 0.001 *n*^2^ = 0.22
RAVLT delayed recall	5.19 (4.10)	10.06 (3.19)	4.69 (3.36)	2.05 (2.49)	*P* < 0.001 *n*^2^ = 0.41
RAVLT learning	33.60 (11.86)	47.71 (9.86)	31.95 (9.57)	25.13 (7.48)	*P* < 0,001 *n*^2^ = 0.40
*Biological markers*					
Aβ42% positive,	32.40	8.33	31.27	58.16	*P* < 0.001 Cohen’s W = 0.12
P-tau % positive,	31.07	7.58	30.52	54.61	*P* < 0.001 Cohen’s W = 0.10
MTA % positive,	30.03	12.88	31.51	41.84	*P* < 0.001 Cohen’s W = 0.14

Values in parentheses refer to standard deviation (*SD*). One-way ANOVAs and *t*-tests were performed to test for group differences

*SCI* subjective cognitive impairment, *MCI* mild cognitive impairment, *AD* Alzheimer’s Disease, *Aβ42* Amyloid Beta 42 peptides, *P-tau* phosphorylated tau, *MTA* medial temporal lobe atrophy

*P* = statistical significance values. *N*^2^ = Eta squared

### Regression analyses

#### Episodic memory delayed free recall

**Fig. 1:**
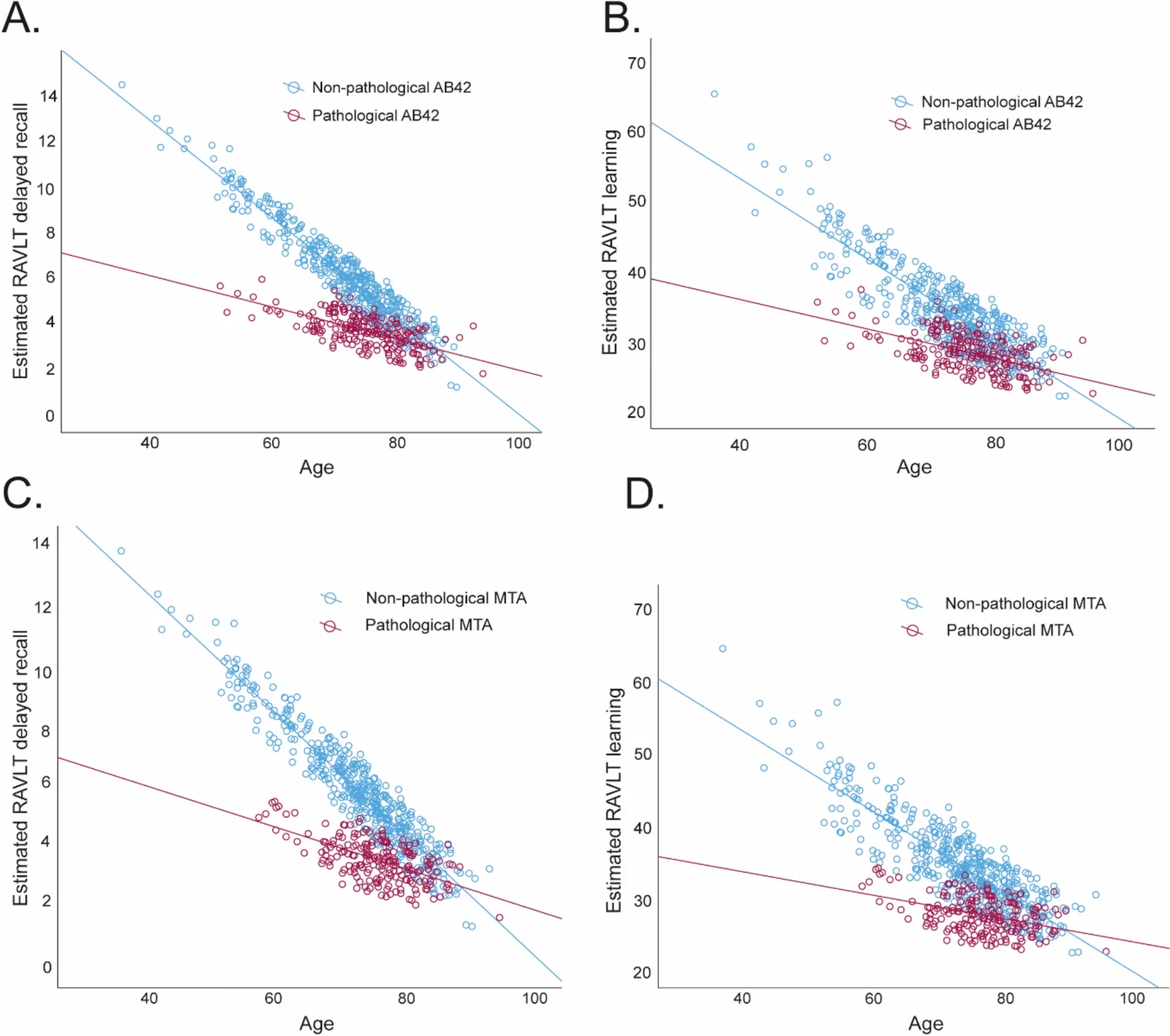
Interaction analyses between age and biomarkers on RAVLT performance. **A** Illustrate the interaction between age and Aβ42 on RAVLT delayed recall performance. **B** Illustrate the interaction between age and Aβ42 on RAVLT learning performance. **C** Illustrate the interaction between age and MTA on RAVLT delayed recall performance. **D** Illustrate the interaction between age and MTA on RAVLT learning performance. The RAVLT values represent the unstandardized predicted values, i.e., the expected value of the RAVLT delayed recall and RAVLT learning (dependent variables) in the regression model for biomarkers and age, without any standardization or scaling applied. The other independent variables were kept constant. *Aβ42* Beta-amyloid. 42, *MTA* medial temporal lobe atrophy, *RAVLT* Rey Auditory Verbal Learning Test

A significant age × Aβ42 interaction was observed, indicating that the negative impact of Aβ42 abnormality on RAVLT delayed recall decreased with increasing age (*β* = 0.14, *p* < 0.001). Similarly, the age × MTA interaction indicated a reduced MTA impact with increasing age (*β* = 0.13, *p* = 0.002). Both models explained 22% of the variance in RAVLT delayed recall (Adjusted R-squared = 0.22, *p* < 0.001). P-tau did not interact significantly with age after the statistical correction (*β* = 0.08, *p* = 0.043). See Table 2A–B regarding the significant interaction effects, Fig. 1 for illustrations, and Supplementary Table 1 for the p-tau interaction result. To verify robustness, we performed the regression analyses using continuous biomarker values. These supplementary analyses confirmed that Aβ42 interacted significantly with age (*β* = − 0.05, *p* = 0.003), and that MTA interacted significantly with age (*β* = 0.08, *p* < 0.001; see Supplementary Table 2). Including memory clinic sites, brain imaging methods (CT or MRI), and CSF analysis methods in the statistical model did not alter significant interactions, maintaining *p* < 0.001.

**Table 2:** Coefficients in regression analyses of age and biomarkers on episodic memory performance

	Unstandardized Coefficients		*t*	Significance	95.0% Confidence Interval for B	
	B	Std. error			Lower bound	Upper bound
*Aβ42 x age interaction on RAVLT delayed recall*						
(Constant)	19.22	1.63	11.80	< 0.001	16.02	22.42
Gender	−0.82	0.27	−2.99	0.003	−1.36	−0.28
Years of Education	0.13	0.04	3.36	< 0.001	0.05	0.20
Age at testing	−0.19	0.02	−9.69	< 0.001	−0.23	−0.15
Aβ42, pathological or not	−1.98	0.30	−6.49	< 0.001	−2.57	−1.38
Aβ42 x Age interaction	0.13	0.04	3.07	0.002	0.05	0.21
*MTA x age interaction on RAVLT delayed recall*						
(Constant)	19.22	1.63	11.80	< 0.001	16.02	22.42
Gender	−0.82	0.27	−2.99	0.003	−1.36	−0.28
Years of Education	0.13	0.04	3.36	< 0.001	0.05	0.20
Age at testing	−0.19	0.02	−9.69	< 0.001	−0.23	−0.15
MTA, pathological or not	−1.98	0.30	−6.49	< 0.001	−2.57	−1.38
MTA x Age interaction	0.13	0.04	3.07	0.002	0.05	0.21

*Aβ42* Beta-amyloid. 42, *MTA* medial temporal lobe atrophy, *t* *t*-values, *B* beta values

The statistical threshold was 0.008

#### Episodic memory learning (encoding)

No significant interaction effects of age and the biomarkers regarding the association with RAVLT learning were shown. See supplementary Table 2 regarding the coefficients.

#### Age cut-offs

**Fig. 2:**
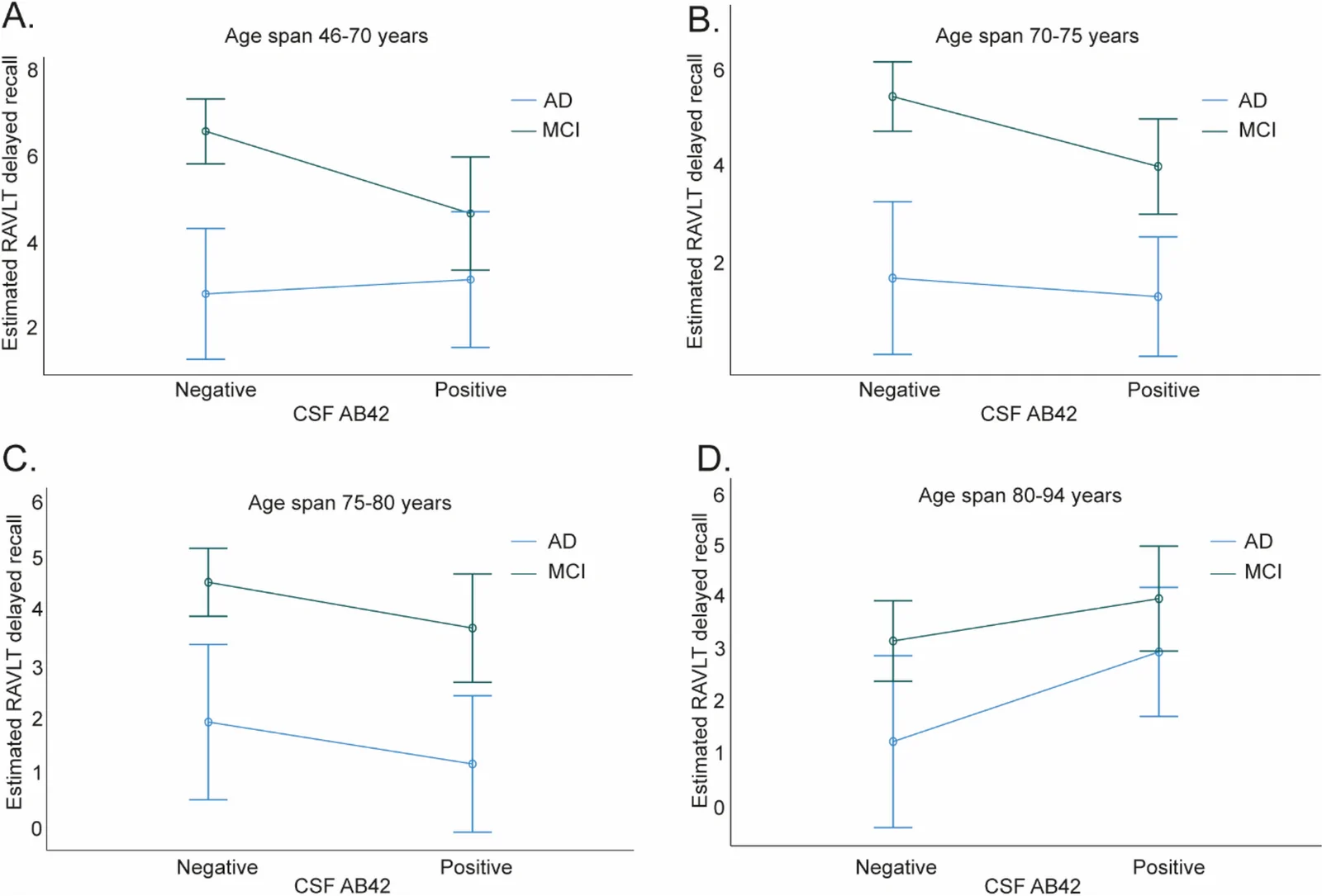
Age spans in the MCI and AD groups. Illustrate the interactions between age and Aβ42 on RAVLT delayed recall performance. **A** AD = 14 with positive Aβ42 marker and 15 with negative. MCI = 20 with a positive Aβ42 marker and 61 with a negative. **B** AD = 23 with positive Aβ42 marker and 14 with negative. MCI = 36 with a positive Aβ42 marker and 68 with a negative. **C** AD = 22 with positive Aβ42 marker and 17 with negative. MCI = 35 with a positive Aβ42 marker and 89 with a negative. **D** AD = 23 with positive Aβ42 marker and 13 with negative. MCI = 35 with a positive Aβ42 marker and 59 with a negative. *AD* Alzheimer’s disease, *MCI* mild cognitive impairment, *CSF* cerebrospinal fluid, *RAVLT* Rey Auditory Verbal Learning Test

The interaction analyses indicated that the effect of ATN on episodic memory weakened with increased age. We were, therefore, interested in exploring more specifically at which ages this attenuation begins. To visualize the interaction effect in diagnostic groups (MCI and AD), we plotted the effect based on different age spans. We observed that for individuals aged 80 and above, the status of Aβ42 (normal or abnormal) did not affect their episodic memory performance (Fig. 2). We also noted a similar pattern for the younger (< 70 years) AD patients, while Aβ42 was associated with episodic memory performance in the MCI patients. Plotting SCI patients was not meaningful because we had only 11 Aβ42-positive patients in total.

To more precisely examine and quantify the age at which abnormal Aβ42 weakens its associations with episodic memory performance, the relationship between abnormal Aβ42 status and episodic memory performance was regressed multiple times, stratified by age. To balance statistical power with the precision of potential age cut-offs, age was stratified into two-year spans. For participants aged 80–82 years and older, abnormal Aβ42 showed a weakened association with episodic memory performance (i.e., *p*-value > 0.36), while it was still associated with episodic memory performance in the younger age spans (Table 3). Thus, negative coefficients of similar magnitude were observed between ages 74 and 80 years, whereas the coefficients were reduced and closer to zero in the age groups above 80 years.

**Table 3:** Abnormal Aβ42 and MTA status being regressed on episodic memory for different age strata

Age span (years)	B	SE	p	N
*Abnormal Aβ42 status being regressed on episodic memory for different age strata*				
74–76	−0.272	0.815	0.014	78
76–78	−0.244	0.807	0.039	80
78–80	−0.290	0.930	0.033	61
80–82	0.133	0.903	0.363	56
82–84	0.136	1.272	0.435	40
*Abnormal MTA status being regressed on episodic memory for different age strata*				
74–76	−0.310	0.466	0.011	67
76–78	−0.369	0.410	0.002	71
78–80	−0.297	0.586	0.044	53
80–82	−0.194	0.599	0.216	50
82–84	0.037	0.829	0.854	32

To balance statistical power with the precision of potential age cut-offs, age was stratified by two-year spans. At 80–82 years and older, abnormal (A) Aβ42 and (B) MTA were no longer associated with episodic memory performance, as indicated by a p-value greater than 0.363, respectively, 0.216

*B* Standardized beta values, *SE* standard error, *p* statistical significance values, *N* number of participants

A similar pattern was also observed for MTA, illustrating that for participants aged 80–82 years and older, abnormal MTA showed a weakened association with episodic memory performance (Table 3).

### Sensitivity analysis

In a sensitivity analysis comparing complete cases with participants who lacked data on CSF biomarkers, RAVLT, and other key variables (i.e., not included) no significant differences were found in global cognitive status (MMSE), gender distribution or years of education across diagnostic groups. However, significant age differences were observed. In the dementia group, included participants were younger than excluded participants (74.48 vs. 79.33 years). Conversely, included participants in the MCI and SCI groups were older than excluded participants (77.29 vs. 74.69 years and 70.63 vs. 66.46 years, respectively).

## Discussion

We evaluated the moderating effect of age on the associations between different ATN measures (Aβ42, p-tau, and MTA) and verbal episodic memory. As hypothesized, we found significant interaction effects of age between A (Aβ42) and N (MTA) on verbal episodic memory recall. Specifically, the negative impact of Aβ42 and MTA abnormalities on episodic memory recall decreased with increasing age. Similar results were also observed for RAVLT learning, though those interactions did not reach significance. These findings indicate a gradual attenuation of the association between biomarkers and cognition with increasing age, reflected by progressively smaller regression coefficients across older age groups. Contrary to our hypothesis, P-tau did not significantly interact with age regarding the episodic memory measures, although the pattern was borderline significant for RAVLT delayed recall. Overall, a similar pattern was evident for all ATN biomarkers. In an exploratory analysis, it is indicated that at age ≥ 80 years, the ATN biomarkers attenuate their associations with performance on verbal learning and episodic memory free recall even more. Moreover, a similar pattern was evident for AD patients aged ≤ 70 years. Our secondary analysis has an exploratory nature and aims to illustrate the age at which the association between verbal learning and episodic memory (free recall) declines further. There are, of course, certain methodological limitations, such as the small number of participants within each age range, and cross-sectional design. Given the exploratory nature of this finding, it should be interpreted with caution, and further longitudinal validation studies are warranted. In addition, this observation should not be interpreted as questioning the broader clinical utility of CSF biomarkers, which may remain important for diagnostic evaluation, prognostic purposes, and determining eligibility for emerging disease-modifying therapies, including anti-amyloid treatments.

Taken together, these findings are partly aligned with previous reports, which show that the diagnostic use of biomarkers is less informative in individuals aged 85 years or older [9, 31], and that age may have distinct effects on the diagnostic performance of AD biomarkers [32]. However, we demonstrate in a naturalistic cohort that the ATN biomarkers showed decreased associations to episodic memory performance in the cognitive impairment continuum, which seems to accelerate with increasing age. The interaction effects in the current study were not unexpected. ATN biomarkers, such as Aβ42, begin to accumulate well before the onset of clinical symptoms, and abnormal Aβ42 values have been detected in up to 40% of healthy elderly individuals [33, 34]. Furthermore, when cognitive symptoms ultimately appear, Aβ42 levels have been mainly saturated, at least partly explaining the low correlation with cognitive decline [35]. So, apart from the expected interaction effects, from a clinical diagnostic perspective, it is unexpected that the interaction lines started to cross at such a relatively young age [9, 10]. The characteristics of the memory clinic population may explain some of the findings, because we used a relatively large number of participants with unselected inclusion criteria, representing a naturalistic heterogeneous population. Aging and AD pathology are heterogeneously processed [36], and with advancing age, the link between AD-type pathology and dementia weakens [37], while other pathologies, such as vascular and inflammatory, become more prominent. The clinical “90+ Study” illustrates this, showing that half of the participants without dementia met AD pathological criteria [10]. Clinical populations may reflect this heterogeneity more than more selected research cohorts [35], as diagnosis primarily reflects the clinical implications rather than the ATN markers. Importantly, despite methodological challenges, the present finding raises the interesting question of whether CSF assessment is justified in patients older than 80 years, given its weak associations with core clinical measures, such as episodic memory.

MTA often correlates with hippocampal volumes and is valid in separating AD from healthy controls [38]. Compared to Aβ42, MTA ratings are generally more available and widely used in clinical settings [39]. MTA is not as AD-specific as Aβ42, and the sensitivity and specificity vary significantly when identifying AD in relation to other causes of dementia [39]. However, in the present study, MTA aligned with Aβ42 and demonstrated a corresponding pattern regarding the negative impact on RAVLT delayed recall, which was diminished with increasing age. This finding is noteworthy because MTA is generally regarded as a marker of neurodegeneration that is more proximally linked to cognitive impairment than amyloid pathology. One might, therefore, expect MTA to maintain its predictive value even at advanced ages. In the oldest old, medial temporal lobe atrophy may become less specific for Alzheimer-related neurodegeneration and increasingly reflect a mixture of age-related processes and comorbid pathologies. Moreover, cognitive performance in this age group is likely influenced by a broader range of factors, including vascular disease, hippocampal sclerosis, TDP-43 pathology, and individual differences in cognitive reserve. Given the exploratory nature of this finding, it should be interpreted with caution and confirmed in independent studies.

A secondary result was the absence of association between Aβ42 and episodic memory performance in younger AD patients (< 70 years). However, given the small sample size (15 Aβ42-negative and 14 Aβ42-positive individuals), it is unclear whether this finding reflects limited statistical power or a true lack of association between Aβ42 and cognitive performance. Therefore, these results should be cautiously interpreted and viewed as exploratory. One possible explanation is that younger patients may constitute a more heterogeneous clinical group, although this hypothesis cannot be evaluated using the present data and requires investigation in larger longitudinal cohorts [9].

Both Aβ42 and MTA, but not p-tau, exhibited significant interactions with age in relation to delayed memory recall. Nevertheless, all three biomarkers demonstrated a similar overall pattern, although the association was comparatively weaker for p-tau. This may indicate that the association between p-tau and episodic memory is less modified by age compared with Aβ42 and MTA, although the absence of a significant interaction should not be interpreted as evidence that p-tau is entirely age-independent. Previous literature indicates that the presence of p-tau might not be as strongly related to age as Aβ42 and total tau [40]. P-tau has also, unlike total tau, long been interpreted as a primarily specified AD biomarker [34]. This, along with the findings from the current clinical sample regarding the effect of p-tau on episodic memory, supports the interaction of p-tau as a relatively AD-specific biomarker whose relationship with episodic memory may be less influenced by age than that of Aβ42 and MTA. However, although rare, in a clinical context, abnormal p-tau levels without amyloidosis are not associated with cognitive deterioration compared to healthy controls [41].

This study has both strengths and limitations. We emphasize that alternative explanations for the observed age-related attenuation should also be considered, including measurement effects, selection bias, age-related comorbidity, and survivorship. Because of the cross-sectional design, the present study cannot determine the extent to which these factors contributed to the observed associations. We acknowledge that participants excluded due to missing age data were older than those included in the analyses. Since our findings suggest that biomarker effects diminish with increasing age, the exclusion of older individuals may have influenced the estimated interaction effects. Specifically, the analyzed sample may underrepresent individuals in whom biomarker associations are weaker. At the same time, these findings likely reflect the fact that it is more common to perform CSF assessments in memory clinic patients when they are younger and diagnostically more challenging. In that sense, our sample is representative. To illustrate age-related changes, we used two-year age intervals. These relatively small groups increase the risk of type 1 error; therefore, these findings should be interpreted with caution. In that sense, the findings might be considered hypothesis-generating rather than confirmatory. Although the use of a linear model for data exhibiting a floor effect has inherent limitations, the weighted least squares (WLS) approach partly addresses this issue by assigning observation-specific weights. Furthermore, bias arising from the floor effect is unlikely to inflate the interaction coefficient, as the model does not produce negative predicted RAVLT scores, indicating that predictions remain within the plausible range of the outcome variable (see Fig. 1).

We examined the relationship between ATN biomarkers and episodic memory across different age groups in a real-world setting using a naturalistic cohort. This design enhances generalizability to patients with early cognitive impairment and dementia, but limits control over comorbidities, imaging methods, or biomarker procedures compared to selective cohorts. All patients received a clinical diagnosis, with ATN biomarkers being one of several factors considered. Data were collected during routine assessments across nine memory clinics, so full standardization was not possible. However, factors, such as hearing impairment, comorbidities, and medication, use were considered in the diagnostic process. We evaluated biomarkers individually rather than in combination, which limits capturing the full spectrum of AD-related abnormalities. Measures, such as MTA, can reflect both AD and normal aging, making interaction effects likely, with age amplifying their impact on episodic memory.

The etiology of SCI and MCI remains largely unknown, highlighting the need for longitudinal follow-up to understand progression. Some circular bias may exist in the subgroup analyses due to reliance on cognitive tests and ATN, though diagnoses were based on comprehensive evaluations, not solely on the assessments included here. Another limitation is that visual ratings were based on either MRI (40%) or CT (60%). However, the inter-rater reliability is comparable to the consistency observed with MRI over time [28, 42]. We focused on verbal learning and free recall of episodic memory content. Future studies should also assess measures of memory recognition or cued delayed recall. Future studies should expand the scope and explore other cognitive domains, such as executive functions related to biomarkers, and their sensitivity in older ages. In addition, a larger sample should have enabled further definition of MCI, i.e., single/multi-domains, and amnestic/non-amnestic characteristics.

## Conclusions

We reported interaction effects between age and biomarkers (Aβ42 and MTA) on verbal episodic memory recall. Specifically, the negative impact of Aβ42 and MTA abnormalities on episodic memory recall diminished with increasing age. P-tau showed a similar pattern, but did not significantly interact with age in relation to the episodic memory measures. In a naturalistic memory clinic setting, we demonstrated that the ATN biomarkers attenuate their association with performance on core clinical measures of verbal episodic memory with increasing age, and in an exploratory analysis that this association seems to weaken even more after the age of 80. We conclude that the ATN biomarkers may weaken their diagnostic sensitivity earlier than previously indicated. The clinical population and naturalistic nature of our study may enhance the explanations of our findings. However, large-scale longitudinal clinical studies are still needed to validate these results.

## Supplementary Information

Below is the link to the electronic supplementary material.

Supplementary file1 (DOCX 335 KB)

Supplementary file2 (DOCX 22 KB)

Supplementary file3 (DOCX 18 KB)

## Data Availability

The data may be made available upon reasonable request, provided that legal and ethical requirements are met.
