# Supplementary material for: The associations between ATN biomarkers and episodic memory diminish as age increases
Source: Eur Geriatr Med. 2026 Sep 8;17(5):2557–68. doi: 10.1007/s41999-026-01606-8 (PMC13754963; doi:10.1007/s41999-026-01606-8)
Supplement: Supplementary file 1 [file 41999_2026_1606_MOESM1_ESM.docx]

**Supplementary Figure**

Illustration of (A) Aβ42 levels and (B) P-tau levels for patients with a clinical AD diagnosis. The Enzyme-linked immunosorbent assay (ELISA) method was used to analyze the CSF data, and the cut-off for Aβ42 abnormality was set at < 550 ng/L, and the cut-off for P-tau abnormality was set at > 80 ng/L. Blue represents AB42 abnormalities, and orange represents Aβ42 normality. The same pattern is valid for P-tau, i.e., blue represents abnormalities, and orange represents normality. X-axis = patients. Y-axis = Nanograms per litre (ng/l).
