# Supplementary material for: The associations between ATN biomarkers and episodic memory diminish as age increases
Source: Eur Geriatr Med. 2026 Sep 8;17(5):2557–68. doi: 10.1007/s41999-026-01606-8 (PMC13754963; doi:10.1007/s41999-026-01606-8)
Supplement: Supplementary file 2 [file 41999_2026_1606_MOESM2_ESM.docx]

| **Dependent variable: RAVLT learning** | **Unstandardized Coefficients** | | **t** | **Significance** | **95.0% Confidence Interval for B** | |
| --- | --- | --- | --- | --- | --- | --- |
|  | **B** | **Std. error** |  |  | **Lower bound** | **Upper bound** |
| **(Constant)** | 1.857 | .062 | 30.123 | <.001 | 1,736 | 1,978 |
| **Gender** | .061 | .011 | 5.522 | <.001 | ,040 | ,083 |
| **Years of Education** | .006 | .002 | 4.062 | <.001 | ,003 | ,009 |
| **Age at testing** | -.006 | .001 | -8.263 | <.001 | -,008 | -,005 |
| **AB42, pathological or not** | -.058 | .012 | -4.783 | <.001 | -,082 | -,034 |
| **AB42 x Age**  **interaction** | .003 | .002 | 2.186 | .029 | ,000 | ,007 |

**Supplementary Table 1. Coefficients from regression analyses of age and biomarkers on episodic memory performance**

**AB42 x age interaction on RAVLT learning**

**MTA x age interaction on RAVLT learning**

| **Dependent variable: RAVLT learning** | **Unstandardized Coefficients** | | **t** | **Significance** | **95.0% Confidence Interval for B** | |
| --- | --- | --- | --- | --- | --- | --- |
| **Learning** | **B** | **Std. error** |  |  | **Lower**  **bound** | **Upper**  **bound** |
| **(Constant)** | 1,941 | ,061 | 32,052 | <,001 | 1,822 | 2,060 |
| **Gender** | -,057 | ,011 | -5,113 | <,001 | -,078 | -,035 |
| **Years of Education** | ,007 | ,002 | 4,680 | <,001 | ,004 | ,010 |
| **Age at testing** | -,006 | ,001 | -8,090 | <,001 | -,007 | -,004 |
| **MTA, pathological or not** | -,075 | ,013 | -5,952 | <,001 | -,100 | -,050 |
| **MTA x Age**  **interaction** | ,004 | ,002 | 2,199 | ,028 | ,000 | ,007 |

**p-tau x age interaction on RAVLT learning**

| **Dependent variable: RAVLT learning** | **Unstandardized Coefficients** | | **t** | **Significance** | **95.0% Confidence Interval for B** | |
| --- | --- | --- | --- | --- | --- | --- |
|  | **B** | **Std. error** |  |  | **Lower**  **bound** | **Upper**  **bound** |
| **(Constant)** | 1,965 | ,063 | 31,380 | <,001 | 1,842 | 2,088 |
| **Gender** | -,068 | ,011 | -6,084 | <,001 | -,090 | -,046 |
| **Years of Education** | ,006 | ,002 | 4,117 | <,001 | ,003 | ,009 |
| **Age at testing** | -,052 | ,012 | -4,275 | <,001 | -,075 | -,028 |
| **P-tau, pathological or not** | -,006 | ,001 | -7,818 | <,001 | -,007 | -,004 |
| **P-tau x Age**  **interaction** | ,001 | ,002 | ,882 | ,378 | -,002 | ,004 |

**P-tau x age interaction on RAVLT delayed recall**

| **Dependent variable: RAVLT delayed recall** | **Unstandardized Coefficients** | | **t** | **Significance** | **95.0% Confidence Interval for B** | |
| --- | --- | --- | --- | --- | --- | --- |
| **Delayed** | **B** | **Std. error** |  |  | **Lower**  **bound** | **Upper**  **bound** |
| **(Constant)** | 19,803 | 1,667 | 11,877 | <,001 | 16,529 | 23,077 |
| **Gender** | -1,121 | ,273 | -4,104 | <,001 | -1,657 | -,585 |
| **Years of Education** | ,108 | ,037 | 2,893 | ,004 | ,035 | ,181 |
| **Age at testing** | -1,749 | ,293 | -5,967 | <,001 | -2,325 | -1,174 |
| **P-tau, pathological or not** | -,189 | ,020 | -9,361 | <,001 | -,229 | -,150 |
| **P-tau x Age**  **interaction** | ,077 | ,038 | 2,026 | ,043 | ,002 | ,152 |

AB42 = Beta-amyloid. 42. MTA = medial temporal lobe atrophy. P-tau = phosphorylated tau. t = t-values. B = beta values. The statistical threshold was 0.008.
