# Supplementary material for: The associations between ATN biomarkers and episodic memory diminish as age increases
Source: Eur Geriatr Med. 2026 Sep 8;17(5):2557–68. doi: 10.1007/s41999-026-01606-8 (PMC13754963; doi:10.1007/s41999-026-01606-8)
Supplement: Supplementary file 3 [file 41999_2026_1606_MOESM3_ESM.docx]

**Supplementary Table 2: Coefficients from WLS Regression analyses with biomarkers as continuous variables**

**AB42 x age on RAVLT delayed recall**

**MTA x age on RAVLT delayed recall**

| **Dependent variable: RAVLT delayed recall** | **Unstandardized Coefficients** | | **t** | **Significance** | **95.0% Confidence Interval for B** | |
| --- | --- | --- | --- | --- | --- | --- |
|  | **B** | **Std. error** |  |  | **Lower**  **bound** | **Upper**  **bound** |
| **(Constant)** | 15,631 | 1,497 | 10,441 | <,001 | 12,691 | 18,571 |
| **Gender** | -1,015 | ,273 | -3,717 | <,001 | -1,551 | -,479 |
| **Years of Education** | ,094 | ,037 | 2,542 | ,011 | ,021 | ,167 |
| **Age at testing** | -,141 | ,018 | -7,998 | <,001 | -,175 | -,106 |
| **AB42, pathological or not** | 1,013 | ,146 | 6,913 | <,001 | ,725 | 1,300 |
| **AB42 x Age**  **interaction** | -,052 | ,017 | -3,010 | ,003 | -,086 | -,018 |

| **Dependent variable: RAVLT delayed recall** | **Unstandardized Coefficients** | | **t** | **Significance** | **95.0% Confidence Interval for B** | |
| --- | --- | --- | --- | --- | --- | --- |
|  | **B** | **Std. error** |  |  | **Lower**  **bound** | **Upper**  **bound** |
| **(Constant)** | 19,22 | 1,63 | 11,80 | <,001 | 16,02 | 22,42 |
| **Gender** | -,82 | ,27 | -2,99 | ,003 | -1,36 | -,28 |
| **Years of Education** | ,13 | ,04 | 3,36 | <,001 | ,05 | ,20 |
| **Age at testing** | -,19 | ,02 | -9,69 | <,001 | -,23 | -,15 |
| **MTA, pathological or not** | -1,98 | ,30 | -6,49 | <,001 | -2,57 | -1,38 |
| **MTA x Age**  **interaction** | ,13 | ,04 | 3,07 | ,002 | ,05 | ,21 |

AB42 = Beta-amyloid. 42. MTA = medial temporal lobe atrophy. t = t-values. B = beta values. The statistical threshold was 0.008.
